# Supplementary figures and images for: The disappearing hand: vestibular stimulation does not improve hand localisation
Source: PeerJ. 2019 Jul 26;7:e7201. doi: 10.7717/peerj.7201 (PMC6662564; doi:10.7717/peerj.7201)

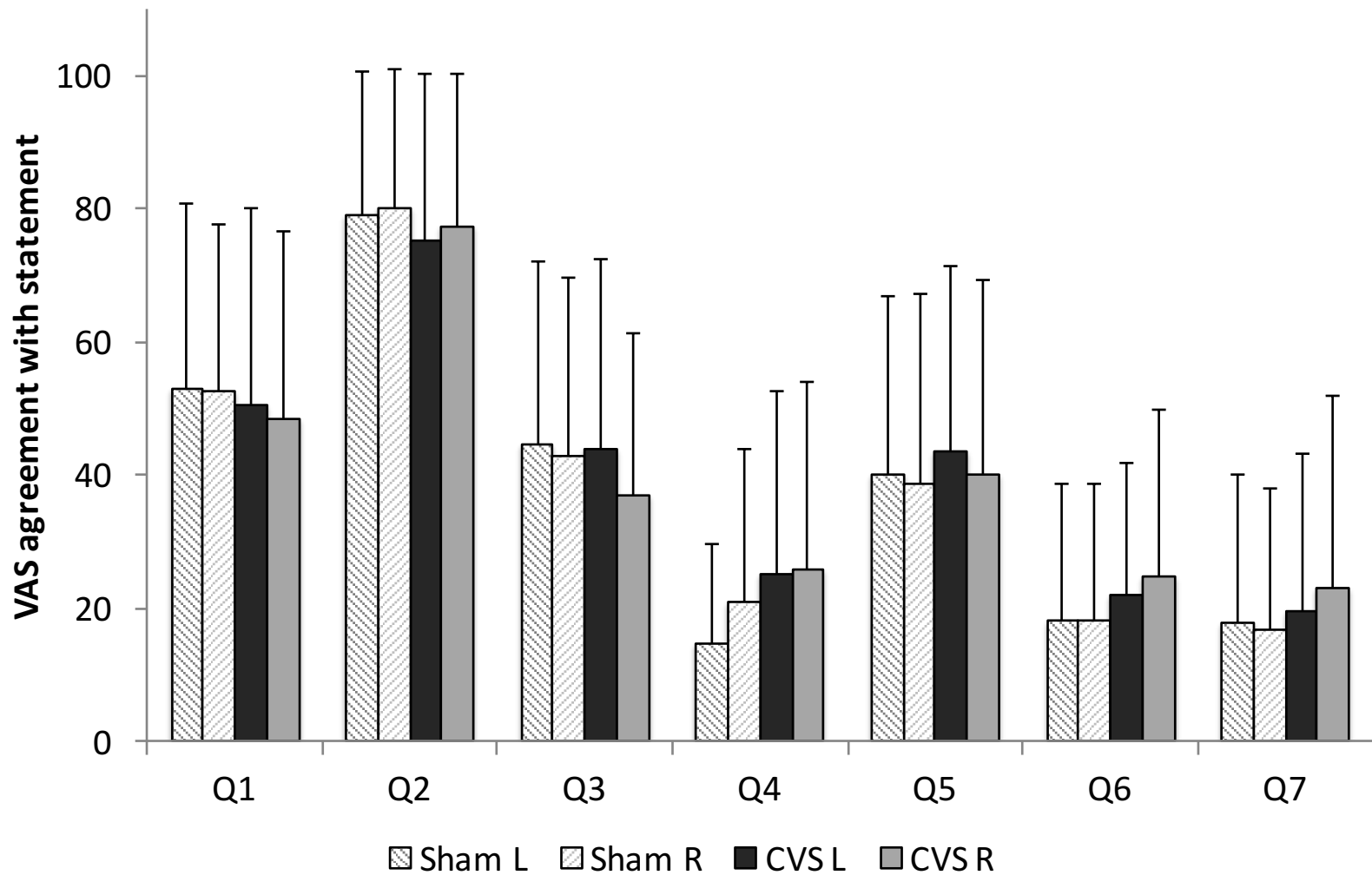

Supplement: Supplemental Information 1 — Mean and standard deviation (error bars) for responses to the questions completed after each round of MIRAGE testing. VAS is a 100 mm visual analogue scale ranging from ‘don’t agree at all’ at 0 to ‘perfectly agree’ at 100 mm. Please refer to Table 1 for text of the questions asked. [file peerj-07-7201-s001.pdf]

# Symptoms with CVS

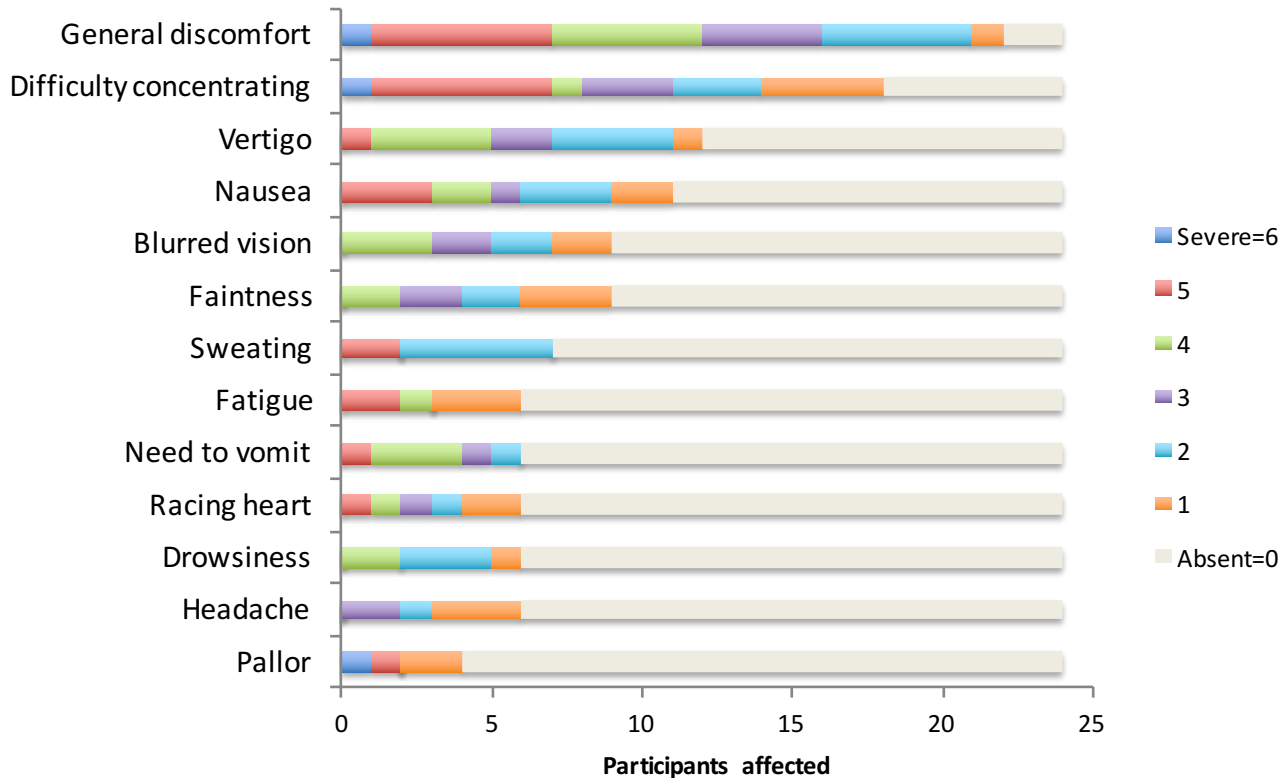

Supplement: Supplemental Information 2 — Number of participants and with what intensity each item was experienced during CVS. [file peerj-07-7201-s002.pdf]

## Symptoms with sham

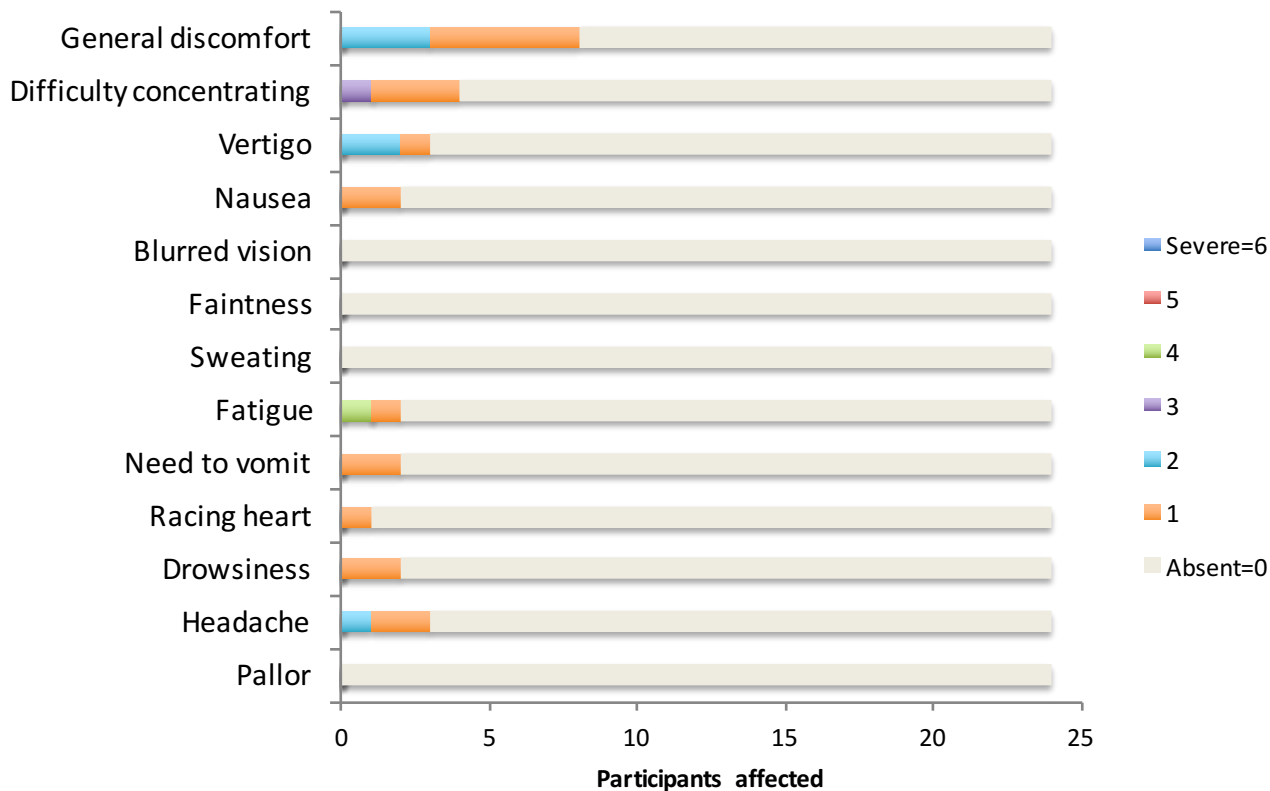

Supplement: Supplemental Information 3 — Number of participants and with what intensity each item was experienced during sham. [file peerj-07-7201-s003.pdf]
